# The Effect of Heat-Killed Candida Albicans and Dentin Powder on the Antibacterial Activity of Chlorhexidine Solution

**Published:** 2012-06-01

**Authors:** Zahed Mohammadi, Sousan Shalavi

**Affiliations:** 1. Department of Endodontics, Hamedan University of Medical Sciences, Hamedan and Iranian Center for Endodontic Research, Tehran, Iran; 2. Dental Student, Hamadan University of Medical Sciences, Hamedan, Iran

**Keywords:** Candida Albicans, Chlorhexidine, Dentin, Heat-Killed, Inactivation, Temperature

## Abstract

**Introduction:**

The purpose of this study was to compare the inhibitory effect of heat-killed Candida albicans and dentin powder on the antibacterial activity of chlorhexidine (CHX) against Enterococcus faecalis and Streptococcus sanguis.

**Materials and Methods:**

The antibacterial effect of each group was determined by measuring the zone of inhibition diameter in millimeters after incubation at 37°C for 24 hours in a humid atmosphere. Each test was repeated three times. Data were analyzed using ANOVA and Tukey’s test.

**Results:**

Results indicated that both heat-killed C. albicans and dentin powder decreased the antibacterial activity of CHX against both tested bacteria significantly (P<0.05).

**Conclusion:**

In conclusion, both heat-killed Candida albicans and dentin powder reduced the antibacterial activity of CHX significantly.

## Introduction

The major role of microorganisms in the pathogenesis of pulp and periapical diseases has clearly been demonstrated [1][2][3]. The elimination of microorganisms from infected root canal systems is a complicated task involving the use of various instrumentation techniques, irrigation regimens and intra-canal medicaments [4][5]. In vitro and in vivo evidence has shown that mechanical instrumentation leaves significant portions of the root canal walls untouched [6] and complete elimination of bacteria by instrumentation alone is unlikely to occur [6]. This has been attributed to the complex anatomy of the root canal system [4][5]. Therefore, a root canal irrigant with antimicrobial activity is required to remove residual tissue and kill microorganisms.

Chlorhexidine (CHX) is a synthetic cationic (positively charged) bis-guanide consisting of two symmetrical 4-cholorophenyl rings and two biguanide groups connected by a central hexamethylene chain. Several studies have confirmed the antibacterial activity of CHX as a root canal irrigation solution [7]. It seems that when CHX and sodium hypochlorite are used at identical concentrations, their antibacterial effects ex vivo (in infected dentine) and in vivo (in the root canal system) are similar [8]. Apart from their antibacterial activity, NaOCl and CHX each have a major advantage. NaOCl possesses the greatest tissue dissolving ability among the root canal irrigants [8]. CHX has a unique property entitled substantivity, which means that CHX molecules attached to hydroxyapatite are released over a long time (approximately 12 weeks) [7].

The inhibitory effect of dentin powder, bovine serum albumin (BSA), hydroxyapatite (HA) and heat-killed microorganisms on the antibacterial activity of CHX has been demonstrated [9][10]. The studies evaluated the effect of the mentioned inhibitors on low concentrations of CHX from 0.01% to 0.5% [11]. It seems that 2% is the best concentration of CHX for root canal irrigation [7]. There is no study on the effect of heat-killed Candida albicans and dentin powder on the antibacterial activity of 2% CHX solution. Therefore, we decided to assess the effect of heat-killed Candida albicans and dentin powder on the antibacterial activity of 2% CHX solution using the agar diffusion test (ADT).

## Materials and Methods

The materials used in the present study were CHX (Clorohexidina Lacer, Barcelona, Spain), dentin powder and heat-killed C. albicans.

Dentin powder was prepared as follows: human third molars were extracted and kept in 0.5% sodium hypochlorite to remove soft tissue and prevent bacterial growth. Before further preparation, the teeth were rinsed and autoclaved (121˚C, 15 min) in an excess of distilled water to remove sodium hypochlorite from the root canal system. The crowns of the teeth were removed with a diamond saw (Accutom, Struers, Denmark) and the roots were crushed between two clean metal blocks. The crushed dentin (particle size 1±4 mm in diameter) was then ground with a shaking apparatus of a marble ball and bowl to obtain dentin powder with a particle size of 0.2±20 µm in diameter. The powder was suspended in distilled water at a concentration of 28 mg per aliquot of 50 µL.

Twenty-eight mg dentin powder and 22 mg heat-killed C. albicans, as inhibitors, were suspended in 50 µL of sterile water. Fifty µL of the inhibitor suspensions were thoroughly mixed and incubated with 50 µL CHX in sealed test tubes at 37 ˚C for 1 hour before being added to 50 µL of the bacterial suspension, giving a total volume of 150 µL. One control group consisted of 50 µL sterile water instead of heat-killed Candida albicans or dentin powder and the other control group consisted of 50 µL of sterile water instead of CHX. The suspensions were carefully mixed and incubated at 37˚C in air.

Overnight cultures of Enterococcus (E.) faecalis (ATCC 12567) and Streptococcus sanguis (ATCC 12487) were used. After growing bacteria in tryptic-soy broth, bacterial suspensions were adjusted to the turbidity of a 0.5 Mc Farland BaSo4 standard (~1.5×10^8^ colony forming unit (CFU) mL^-1^). Fifty Petri dishes containing Tryptic-soy agar enriched with 5% defibrinated sheep blood and supplemented with Hemin and vitamin K were seeded with bacteria. Seeding was done using sterile cotton-tipped applicators that were brushed across the agar surface. Three wells 5 mm deep and 6 mm in diameter were punched in each agar plate and filled with freshly mixed materials. Seventy-five wells were used for each group. The plates were then maintained at room temperature for two hours for pre-diffusion of the material.

Later, the antimicrobial effect of each material was determined by measuring the diameter of the zone of inhibition in millimeters after incubation at 37°C for 24 hours in a humid atmosphere. Each test was repeated three times. All the zones of inhibition were measured by one observer.

Data were analyzed using ANOVA and Tukey’s tests. Differences at the 5% level were considered statistically significant.

## Results

None of the control groups showed bacterial growth inhibition, which validated the methodology of the study. All tested materials demonstrated some antibacterial activity. Both heat-killed Candida albicans and dentin powder decreased the efficacy of CHX against both bacteria significantly (P<0.05). The means of the diameters of the zone of microbial inhibition for each group against each bacterium are shown in Table 1.

**Table 1:** Mean diameter and standard deviations of bacterial growth inhibition zones of the three materials (in mm)^a^

**Experimental groups**	**CHX**	**CHX+HKCA**	**CHX+DP**
**Bacterial species**			
*S. sanguis*	18.40±1.87	10.74±1.21	10.78±1.10
*E. faecalis*	15.69±1.59	9.72±0.97	10.21±1.42

^a^ CHX=Chlorhexidine; HKCA=Heat-killed Candida albicans; DP=Dentin powder

## Discussion

Microorganisms are the main etiologic agents in endodontic pathosis [1][2][3]. The agar diffusion method is widely used to assess the antibacterial activity of dental materials [12][13][14]. The contact between the experimental material and agar, molecular weight, size and shape of the antimicrobial agent, load and concentration of the test material, as well as the agar gel viscosity, ionic concentration in relation to the medium, control and standardization of inoculation density, evaluation of results, selection of agar medium, selection of microorganisms are restricting factors affecting the dynamics and variability of diffusion tests in an agar medium [15]. Moreover, the depth of agar medium, incubation temperature of the plates, and the reading point of inhibition haloes are also restricting factors [15]. If most of these variables are carefully controlled, consistent and reproducible results may be achieved [12]. As a result of the obvious limitations of in vitro studies, clinical inferences should be drawn with strict caution.

Enterococcus faecalis was chosen for the following reasons: i) its prevalence in root-filled teeth with periradicular lesions using either culturing and polymerase chain reaction (PCR) methods is 24-70% and 67-77%, respectively; ii) its capacity to endure prolonged periods of starvation until an adequate nutritional supply becomes available has been demonstrated; and iii) it can induce infection separately (as a mono-infection) [16].

The reason for adding bacterial suspension was to put the microbial suspension in contact with the mixture of CHX and hydroxyapatite or the mixture of CHX and bovine serum albumin.

CHX is a positively charged hydrophobic and lipophilic molecule that interacts with phospholipids and lipopolysaccharides on bacterial cell membranes and then enters the cell through some type of active or passive transport mechanism [7]. Its efficacy is due to the interaction of the positive charge of the molecule and the negatively charged phosphate groups on microbial cell walls [7], thereby altering the cells' osmotic equilibrium. This increases the permeability of the cell wall, which then allows the CHX molecule to penetrate into the bacteria. Several studies have proven the antibacterial activity of CHX [7].

In an ex vivo study [17], the effectiveness of calcium hydroxide (Ca(OH)_2_), iodine potassium iodide (IKI) and a CHX solution in disinfecting root canal systems that were infected with Actinomyces israelii was assessed. The root canals were exposed to either IKI, calcium hydroxide or 2% CHX for periods of 3, 7 and 60 days. CHX was the only disinfectant to eliminate A. israelii from all samples at all time periods while 25% of the samples treated with iodine potassium iodide and 50% of the specimens treated with Ca(OH)_2_ still had viable A. israelii after treatment. Oncag et al. evaluated the antibacterial properties of 5.25% sodium hypochlorite (NaOCl), 2% CHX and 0.2% CHX plus 0.2% cetrimide (Cetrexidin (GABA Vebas, San Giuliano Milanese, Italy)) after 5 minutes and 48 hours on extracted human teeth after the canals had been infected by E. faecalis [18]. The 2% CHX and Cetrexidin were significantly more effective on E. faecalis than the 5.25% NaOCl at both time points. Two studies [19][20] investigated the ex vivo antimicrobial activity against endodontic pathogens of three concentrations (0.2%, 1% and 2%) of two forms of CHX (gel and liquid) and compared them with five concentrations of NaOCl (0.5%, 1%, 2.5%, 4% and 5.25%). Both the 2% gel and 2% liquid formulations of CHX eliminated Staphylococcus aureus and Candida albicans within 15 seconds, whereas the gel formulation killed E. faecalis within 1 minute. All of the tested irrigants eliminated Porphyromonas endodontalis, Porphyromonas gingivalis and Prevotella intermedia within 15 seconds. The time required for 1.0% and 2.0% CHX liquid to eliminate all microorganisms was the same as that required for 5.25% NaOCl [19][20].

Several in vivo studies have confirmed the antibacterial activity of CHX. Siqueira et al. found that both 2.5% sodium hypochlorite and 0.12% CHX had comparable effects in eliminating bacteria and they suggested that both could be used as irrigants [21].

A randomised clinical trial [22] demonstrated that the antibacterial efficacies of calcium hydroxide (Ca(OH)_2_), CHX and a mixture of Ca(OH)_2_/CHX were similar.

The root canal milieu is a complex mixture of a variety of organic and inorganic compounds. Hydroxyapatite, the main component of dentin, is the major representative of the inorganic components present. In addition, inflammatory exudate entering the apical root canal in purulent infections is rich in proteins such as albumin. The relative importance of the various organic and inorganic compounds in the inactivation of root canal disinfectants have been studied exclusively [10].

Portenier et al. found that adding dentin powder to 0.05% CHX acetate completely nullified the antibacterial property of CHX during the first 24 hours. However, the findings of the present study showed that dentin powder reduced the antibacterial activity of CHX significantly [9]. This difference could be attributed to the method used to assess antibacterial activity. Portenier et al. used the direct contact method, whereas ADT was used in this study [9].

In another part of this study, the effect of heat-killed C. albicans on the antibacterial activity of CHX was assessed. To avoid interference with the culturing of the test organisms, the fungal cells were heat-killed before the inhibition experiments [10]. Our findings demonstrated that heat-killed C. albicans reduced the antibacterial activity of CHX significantly. The reason to use heat to kill C. albicans cells was that Gram staining and phase contrast microscopic studies have shown that cell lysis do not occur during heat treatment [10]. Portenier et al. showed that killing of E. faecalis by CHX was completely inhibited by heat-killed C. albicans, which is in contrast to the findings of the present study [10]. This can be attributed to the concentration of CHX used. In the present study, 2% concentration of CHX was used, whereas Portenier et al. employed a 0.02% concentration. In another study, Sassone et al. showed that bovine serum albumin had little to no inhibitory effect on the antibacterial activity of CHX and sodium hypochlorite [23].

## Conclusion

Within the limitations of the present study, both heat-killed C. albicans and dentin powder reduced the antibacterial activity of CHX significantly. Considering the above-mentioned limitations of the Agar diffusion test, it is suggested that the inhibitory effect of heat-killed C. albicans and dentin powder should be assessed using direct contact method in future studies.
